# Supplementary material for: The nutritional status of mycetoma affected patients seen at the Mycetoma Research Center, Sudan
Source: PLoS Negl Trop Dis. 2024 Jan 2;18(1):e0011726. doi: 10.1371/journal.pntd.0011726 (PMC10786388; doi:10.1371/journal.pntd.0011726)
Supplement: S1 Table — (DOCX) [file pntd.0011726.s001.docx]

|  |  | Control | | | | | | Cases | | | | | |
| --- | --- | --- | --- | --- | --- | --- | --- | --- | --- | --- | --- | --- | --- |
|  |  | Under  weight | Normal Weight | Over  weight | Obese  I | Obese  II | P-Value | Under  weight | Normal Weight | Over  weight | Obese I | Obese II | P-Value |
| Age  Group | 8 – 26 | 10 | 30 | 6 | 2 | 1 | .006 | 45 | 27 | 3 | 1 | 0 | .006 |
|  | 27 – 45 | 6 | 41 | 26 | 10 | 4 |  | 13 | 37 | 9 | 4 | 2 |  |
|  | 46 – 64 | 0 | 24 | 11 | 3 | 0 |  | 2 | 11 | 7 | 2 | 1 |  |
|  | 65 – 82 | 3 | 1 | 3 | 0 | 0 |  | 4 | 3 | 6 | 2 | 2 |  |
| Sex | Male | 16 | 77 | 35 | 9 | 1 | 0.015 | 44 | 44 | 11 | 3 | 4 | 0.079 |
|  | Female | 3 | 19 | 11 | 6 | 4 |  | 20 | 34 | 14 | 6 | 1 |  |
| Educational  level | Illiterate | 1 | 12 | 6 | 0 | 1 | 0.98 | 12 | 18 | 6 | 1 | 0 | 0.82 |
|  | Khalwa | 1 | 7 | 4 | 0 | 0 |  | 7 | 7 | 3 | 2 | 2 |  |
|  | Primary school | 7 | 21 | 9 | 6 | 2 |  | 35 | 33 | 12 | 3 | 2 |  |
|  | Secondary | 6 | 29 | 14 | 4 | 1 |  | 5 | 16 | 3 | 3 | 1 |  |
|  | University | 4 | 23 | 11 | 2 | 1 |  | 4 | 3 | 1 | 0 | 0 |  |
|  | Post-graduate | 0 | 4 | 2 | 3 | 0 |  | 1 | 1 | 0 | 0 | 0 |  |
| Occupation | Farmer | 1 | 13 | 0 | 0 | 0 | 0.021 | 8 | 8 | 4 | 0 | 1 | .024 |
|  | Shepherd | 1 | 0 | 1 | 0 | 0 |  | 2 | 4 | 0 | 0 | 0 |  |
|  | Worker | 2 | 10 | 4 | 2 | 0 |  | 2 | 5 | 1 | 0 | 0 |  |
|  | Seller | 0 | 8 | 6 | 3 | 0 |  | 2 | 3 | 0 | 0 | 0 |  |
|  | Student | 4 | 14 | 3 | 1 | 0 |  | 19 | 10 | 1 | 0 | 0 |  |
|  | House Wife | 1 | 8 | 8 | 2 | 3 |  | 7 | 23 | 10 | 6 | 1 |  |
|  | Unemployment | 4 | 5 | 3 | 2 | 1 |  | 16 | 16 | 6 | 1 | 0 |  |
|  | Other | 6 | 38 | 21 | 5 | 1 |  | 6 | 9 | 3 | 2 | 3 |  |
|  | Small |  | | | | | | 7 | 9 | 3 | 0 | 0 | 0.418 |
|  | Medium |  |  |  |  |  |  | 12 | 14 | 4 | 2 | 0 |  |
|  | Massive |  |  |  |  |  |  | 40 | 50 | 15 | 7 | 4 |  |
| Meal taken per-day | 1 | 1 | 1 | 0 | 0 | 0 | 0 .083 | 0 | 0 | 0 | 0 | 0 | 0.178 |
|  | 2 | 5 | 37 | 16 | 9 | 3 |  | 22 | 42 | 8 | 4 | 3 |  |
|  | 3 | 13 | 58 | 28 | 6 | 2 |  | 38 | 33 | 15 | 4 | 2 |  |
| Food  amount  taken | Less | 8 | 11 | 5 | 1 | 2 | 0.17 | 18 | 17 | 6 | 2 | 1 | 0.643 |
|  | Same | 10 | 64 | 35 | 11 | 3 |  | 40 | 56 | 17 | 6 | 4 |  |
|  | More | 1 | 21 | 6 | 3 | 0 |  | 6 | 5 | 2 | 1 | 0 |  |
| Appetite  in relation family | Low | 6 | 13 | 5 | 0 | 2 | 0.058 | 21 | 23 | 11 | 3 | 0 | 0.529 |
|  | Moderate | 10 | 66 | 32 | 11 | 3 |  | 39 | 49 | 13 | 5 | 3 |  |
|  | High | 3 | 17 | 9 | 4 | 0 |  | 4 | 6 | 1 | 1 | 2 |  |
| State |  | | | | | | 0.889 |  | | | | | 0.516 |
| Site |  | | | | | |  |  | | | | | 0.6 35 |
| Duration |  | | | | | |  |  | | | | | .0577 |

**S1:** **The dietary intake and habits of the study population according to the BMI classification**.
