# Supplementary material for: The nutritional status of mycetoma affected patients seen at the Mycetoma Research Center, Sudan
Source: PLoS Negl Trop Dis. 2024 Jan 2;18(1):e0011726. doi: 10.1371/journal.pntd.0011726 (PMC10786388; doi:10.1371/journal.pntd.0011726)
Supplement: S2 Table — (DOCX) [file pntd.0011726.s002.docx]

| Food group:  Cereals | | Control | | | | |  | Cases | | | | |  |
| --- | --- | --- | --- | --- | --- | --- | --- | --- | --- | --- | --- | --- | --- |
|  |  | Underweight | Normal Weight | Overweight | Obese I | Obese II | p-value | Underweight | Normal Weight | Overweight | Obese I | Obese II | P-value |
| Bread | Daily | 9 | 56 | 37 | 12 | 4 | .044 | 33 | 47 | 14 | 8 | 3 | 0.157 |
|  | Once/ week | 2 | 12 | 1 | 0 | 0 |  | 6 | 8 | 3 | 0 | 0 |  |
|  | 2-3/week | 8 | 22 | 6 | 3 | 0 |  | 21 | 17 | 6 | 1 | 2 |  |
|  | 2-3 per month | 0 | 1 | 0 | 0 | 0 |  | 0 | 5 | 1 | 0 | 0 |  |
|  | Never | 0 | 5 | 2 | 0 | 1 |  | 4 | 1 | 1 | 0 | 0 |  |
| Kisra | Daily | 9 | 46 | 12 | 5 | 3 | .059 | 39 | 42 | 15 | 5 | 2 | 0.438 |
|  | Once/ week | 3 | 15 | 9 | 4 | 0 |  | 9 | 4 | 3 | 1 | 1 |  |
|  | 2-3/week | 5 | 22 | 15 | 3 | 1 |  | 11 | 17 | 6 | 2 | 2 |  |
|  | 2-3 per month | 2 | 5 | 8 | 1 | 0 |  | 4 | 9 | 1 | 0 | 0 |  |
|  | Never | 0 | 8 | 2 | 2 | 1 |  | 1 | 6 | 0 | 1 | 0 |  |
| Acida | Daily | 6 | 39 | 14 | 3 | 1 | 0.409 | 29 | 40 | 12 | 3 | 1 | 0.159 |
|  | Once/ week | 0 | 19 | 8 | 4 | 0 |  | 15 | 5 | 2 | 1 | 1 |  |
|  | 2-3 week | 7 | 25 | 14 | 5 | 3 |  | 17 | 20 | 8 | 3 | 1 |  |
|  | 2-3 per month | 3 | 9 | 6 | 2 | 1 |  | 1 | 5 | 2 | 1 | 0 |  |
|  | Never | 3 | 4 | 4 | 1 | 0 |  | 2 | 8 | 1 | 1 | 2 |  |
| Gurasa | Daily | 4 | 14 | 4 | 0 | 0 | 0.025 | 16 | 9 | 4 | 1 | 1 | 0.435 |
|  | Once week | 3 | 18 | 10 | 3 | 1 |  | 8 | 22 | 4 | 1 | 0 |  |
|  | 2-3/week | 9 | 33 | 11 | 7 | 0 |  | 19 | 18 | 9 | 1 | 2 |  |
|  | 2-3 per month | 2 | 13 | 11 | 4 | 1 |  | 4 | 18 | 5 | 3 | 1 |  |
|  | Never | 1 | 18 | 10 | 1 | 3 |  | 17 | 11 | 3 | 3 | 1 |  |
| Rice | Daily | 3 | 13 | 2 | 2 | 1 | 0.9 | 5 | 4 | 2 | 0 | 0 | 0.444 |
|  | Once/ week | 3 | 16 | 7 | 4 | 0 |  | 13 | 14 | 11 | 4 | 0 |  |
|  | 2-3/week | 6 | 26 | 18 | 4 | 2 |  | 24 | 22 | 6 | 1 | 4 |  |
|  | 2-3 per month | 4 | 22 | 11 | 3 | 2 |  | 9 | 21 | 4 | 3 | 1 |  |
|  | Never | 3 | 19 | 8 | 2 | 0 |  | 13 | 17 | 2 | 1 | 0 |  |

**S2: Food items consumed by the study population by Body Mass Index classification**

| Food group:  Oil rich foods | | Control | | | | |  | Cases | | | | |  |
| --- | --- | --- | --- | --- | --- | --- | --- | --- | --- | --- | --- | --- | --- |
|  |  | Underweight | Normal Weight | Overweight | Obese I | Obese II | P-Value | Underweight | Normal Weight | Overweight | Obese I | Obese II | P-Value |
| Peanut-oil | Daily | 9 | 70 | 32 | 13 | 3 | 0.253 | 42 | 54 | 17 | 8 | 3 | 0.642 |
|  | Once/ week | 6 | 5 | 3 | 1 | 0 |  | 8 | 5 | 2 | 0 | 0 |  |
|  | 2-3/week | 3 | 12 | 6 | 1 | 1 |  | 7 | 9 | 5 | 0 | 0 |  |
|  | 2-3 per month | 0 | 1 | 1 | 0 | 1 |  | 2 | 3 | 1 | 0 | 0 |  |
|  | Never | 1 | 8 | 4 | 0 | 0 |  | 5 | 7 | 0 | 1 | 2 |  |
| Sesame-oil | Daily | 2 | 18 | 9 | 1 | 2 | 0.447 | 8 | 10 | 3 | 2 | 0 | 0.291 |
|  | Once/ week | 3 | 11 | 3 | 2 | 0 |  | 6 | 4 | 1 | 1 | 0 |  |
|  | 2-3/week | 3 | 11 | 9 | 3 | 0 |  | 12 | 13 | 9 | 1 | 2 |  |
|  | 2-3 per month | 4 | 13 | 11 | 3 | 3 |  | 10 | 19 | 7 | 3 | 1 |  |
|  | Never | 7 | 43 | 14 | 6 | 0 |  | 28 | 32 | 5 | 2 | 2 |  |
| Sunflower-oil | Daily | 4 | 26 | 17 | 5 | 1 | 0.021 | 17 | 19 | 7 | 2 | 1 | 0.987 |
|  | Once/ week | 1 | 2 | 2 | 0 | 1 |  | 2 | 2 | 0 | 1 | 0 |  |
|  | 2-3/week | 0 | 7 | 7 | 3 | 0 |  | 6 | 7 | 6 | 1 | 0 |  |
|  | 2-3 per month | 0 | 7 | 4 | 0 | 0 |  | 4 | 3 | 1 | 0 | 0 |  |
|  | Never | 14 | 54 | 16 | 7 | 3 |  | 35 | 47 | 11 | 5 | 4 |  |
| Ghee | Daily | 0 | 2 | 1 | 1 | 0 | 0.272 | 3 | 3 | 0 | 1 | 0 | 0.927 |
|  | Once/ week | 3 | 10 | 2 | 0 | 0 |  | 5 | 5 | 0 | 0 | 0 |  |
|  | 2-3/week | 2 | 5 | 5 | 1 | 0 |  | 3 | 9 | 3 | 1 | 0 |  |
|  | 2-3 per month | 5 | 20 | 9 | 2 | 3 |  | 9 | 12 | 5 | 1 | 2 |  |
|  | Never | 9 | 59 | 29 | 11 | 2 |  | 44 | 49 | 17 | 6 | 3 |  |
|  |  |  |  |  |  |  |  |  |  |  |  |  |  |

| Food group  Animal foods | | Control | | | | |  | Cases | | | | |  |
| --- | --- | --- | --- | --- | --- | --- | --- | --- | --- | --- | --- | --- | --- |
|  |  | Underweight | Normal Weight | Overweight | Obese I | Obese II | P-Value | Underweight | Normal Weight | Overweight | Obese I | Obese II | P-Value |
| Meat | Daily | 8 | 40 | 28 | 8 | 3 | 0.044 | 20 | 34 | 8 | 5 | 3 | 0.280 |
|  | Once/ week | 1 | 7 | 4 | 2 | 1 |  | 9 | 13 | 3 | 0 | 0 |  |
|  | 2-3/week | 8 | 42 | 8 | 4 | 1 |  | 28 | 25 | 9 | 4 | 2 |  |
|  | 2-3 per month | 2 | 5 | 5 | 1 | 0 |  | 4 | 5 | 4 | 0 | 0 |  |
|  | Never | 0 | 2 | 1 | 0 | 0 |  | 3 | 1 | 1 | 0 | 0 |  |
| Chicken | Daily | 1 | 5 | 4 | 1 | 1 | 0.756 | 2 | 4 | 1 | 1 | 0 | 0.643 |
|  | Once/ week | 6 | 20 | 8 | 2 | 1 |  | 13 | 15 | 2 | 1 | 0 |  |
|  | 2-3/week | 7 | 28 | 18 | 5 | 2 |  | 19 | 23 | 7 | 3 | 4 |  |
|  | 2-3 per month | 0 | 16 | 7 | 5 | 0 |  | 14 | 17 | 6 | 2 | 1 |  |
|  | Never | 5 | 27 | 9 | 2 | 1 |  | 16 | 19 | 9 | 2 | 0 |  |
| Fish | Daily | 0 | 4 | 0 | 1 | 1 | 0.406 | 0 | 2 | 1 | 1 | 0 | 0.548 |
|  | Once/ week | 1 | 10 | 11 | 1 | 0 |  | 10 | 7 | 0 | 1 | 0 |  |
|  | 2-3/week | 8 | 13 | 10 | 2 | 1 |  | 6 | 9 | 2 | 2 | 2 |  |
|  | 2-3 per month | 7 | 41 | 18 | 8 | 1 |  | 20 | 30 | 10 | 3 | 3 |  |
|  | Never | 3 | 28 | 7 | 3 | 2 |  | 28 | 30 | 12 | 2 | 0 |  |
| Eggs | Daily | 1 | 9 | 5 | 1 | 0 | 0.040 | 5 | 2 | 4 | 2 | 0 | 0.860 |
|  | Once/ week | 2 | 12 | 9 | 0 | 1 |  | 10 | 8 | 2 | 0 | 0 |  |
|  | 2-3/week | 6 | 25 | 15 | 10 | 2 |  | 16 | 27 | 7 | 2 | 1 |  |
|  | 2-3 per month | 2 | 28 | 10 | 3 | 1 |  | 13 | 18 | 5 | 3 | 2 |  |
|  | Never | 8 | 22 | 7 | 1 | 1 |  | 20 | 23 | 7 | 2 | 2 |  |

| Food group:  Dairy | | Control | | | | | |  | Cases | | | | |  |
| --- | --- | --- | --- | --- | --- | --- | --- | --- | --- | --- | --- | --- | --- | --- |
|  |  | Underweight | Normal Weight | | Overweight | Obese I | Obese II | P-Value | Underweight | Normal Weight | Overweight | Obese I | Obese II | P-Value |
| Milk | Daily | 13 | | 62 | 32 | 11 | 3 | 0.701 | 46 | 60 | 18 | 8 | 3 | 0.570 |
|  | Once/ week | 1 | | 3 | 2 | 0 | 1 |  | 1 | 2 | 0 | 0 | 0 |  |
|  | 2-3/week | 3 | | 13 | 5 | 0 | 1 |  | 5 | 8 | 3 | 1 | 1 |  |
|  | 2-3 per month | 0 | | 6 | 4 | 1 | 0 |  | 1 | 2 | 0 | 0 | 0 |  |
|  | Never | 2 | | 12 | 3 | 3 | 0 |  | 11 | 6 | 4 | 0 | 1 |  |
| Yoghurt | Daily | 5 | | 26 | 10 | 3 | 1 | 0.355 | 14 | 23 | 7 | 1 | 0 | 0.934 |
|  | Once/ week | 1 | | 7 | 5 | 0 | 1 |  | 11 | 9 | 2 | 1 | 1 |  |
|  | 2-3/week | 4 | | 25 | 21 | 6 | 2 |  | 13 | 15 | 7 | 3 | 3 |  |
|  | 2-3 per month | 2 | | 13 | 3 | 3 | 1 |  | 6 | 11 | 1 | 2 | 0 |  |
|  | Never | 7 | | 25 | 7 | 3 | 0 |  | 20 | 20 | 8 | 2 | 1 |  |
| Cheese | Daily | 4 | | 15 | 6 | 1 | 0 | 0.577 | 5 | 3 | 2 | 2 | 0 | 0.344 |
|  | Once/ week | 2 | | 10 | 2 | 2 | 0 |  | 5 | 7 | 1 | 0 | 0 |  |
|  | 2-3/week | 2 | | 12 | 13 | 2 | 2 |  | 3 | 9 | 4 | 0 | 0 |  |
|  | 2-3 per month | 3 | | 16 | 3 | 4 | 1 |  | 6 | 16 | 2 | 3 | 1 |  |
|  | Never | 8 | | 43 | 22 | 6 | 2 |  | 45 | 43 | 16 | 4 | 4 |  |

| Food group: Legumes | | Control | | | | |  | Cases | | | | | |  |
| --- | --- | --- | --- | --- | --- | --- | --- | --- | --- | --- | --- | --- | --- | --- |
|  |  | Underweight | Normal Weight | Overweight | Obese I | Obese II | P-Value | Underweight | Normal Weight | Overweight | Obese  I | | Obese II | p-Value |
| Faba-beans | Daily | 4 | 30 | 12 | 4 | 0 | 0.668 | 16 | 12 | 4 | | 1 | 0 | 0.348 |
|  | Once/ week | 5 | 10 | 5 | 1 | 0 |  | 11 | 15 | 2 | | 0 | 2 |  |
|  | 2-3/week | 6 | 31 | 23 | 8 | 2 |  | 15 | 31 | 14 | | 2 | 2 |  |
|  | 2-3 per month | 1 | 13 | 1 | 2 | 2 |  | 9 | 9 | 3 | | 4 | 0 |  |
|  | Never | 3 | 12 | 5 | 0 | 1 |  | 13 | 11 | 2 | | 2 | 1 |  |
| Lentils | Daily | 3 | 10 | 3 | 2 | 0 | 0.426 | 8 | 6 | 3 | | 0 | 0 | 0.275 |
|  | Once/ week | 5 | 25 | 11 | 4 | 1 |  | 15 | 19 | 4 | | 2 | 1 |  |
|  | 2-3/week | 8 | 27 | 20 | 5 | 0 |  | 23 | 28 | 9 | | 4 | 2 |  |
|  | 2-3 per month | 0 | 18 | 9 | 3 | 0 |  | 8 | 12 | 6 | | 1 | 1 |  |
|  | Never | 3 | 16 | 3 | 1 | 4 |  | 10 | 13 | 3 | | 2 | 1 |  |
| Peogon-pea | Daily | 0 | 11 | 1 | 1 | 0 | 0.004 | 9 | 4 | 4 | | 0 | 0 | 0.025 |
|  | Once/ week | 6 | 21 | 8 | 0 | 0 |  | 14 | 14 | 2 | | 0 | 1 |  |
|  | 2-3/week | 5 | 19 | 9 | 4 | 1 |  | 13 | 20 | 7 | | 1 | 1 |  |
|  | 2-3 per month | 6 | 33 | 16 | 7 | 2 |  | 17 | 30 | 7 | | 3 | 1 |  |
|  | Never | 2 | 12 | 12 | 3 | 2 |  | 11 | 10 | 5 | | 5 | 2 |  |
| Beans | Daily | 0 | 1 | 1 | 0 | 0 | 0.837 | 2 | 1 | 0 | | 0 | 0 | 0.530 |
|  | Once/ week | 7 | 22 | 12 | 2 | 1 |  | 12 | 11 | 2 | | 1 | 1 |  |
|  | 2-3/week | 4 | 14 | 10 | 4 | 0 |  | 4 | 16 | 4 | | 3 | 0 |  |
|  | 2-3 per month | 4 | 20 | 10 | 6 | 1 |  | 15 | 15 | 10 | | 1 | 4 |  |
|  | Never | 4 | 39 | 13 | 3 | 3 |  | 31 | 35 | 9 | | 4 | 0 |  |
| Ckickpea | Daily | 0 | 0 | 0 | 1 | 0 | 0.807 | 2 | 4 | 1 | | 0 | 0 | 0.43 |
|  | Once/ week | 2 | 10 | 1 | 2 | 0 |  | 7 | 7 | 0 | | 0 | 1 |  |
|  | 2-3/week | 4 | 14 | 9 | 2 | 0 |  | 6 | 12 | 4 | | 2 | 0 |  |
|  | 2-3 per month | 6 | 30 | 18 | 6 | 1 |  | 19 | 26 | 11 | | 6 | 1 |  |
|  | Never | 7 | 42 | 18 | 4 | 4 |  | 30 | 29 | 9 | | 1 | 3 |  |
| Falafil | Daily | 6 | 17 | 8 | 2 | 0 | 0.475 | 5 | 4 | 3 | | 1 | 0 | 0.460 |
|  | Once/ week | 4 | 17 | 6 | 3 | 1 |  | 10 | 15 | 5 | | 1 | 1 |  |
|  | 2-3/week | 4 | 25 | 18 | 5 | 2 |  | 23 | 30 | 10 | | 4 | 2 |  |
|  | 2-3 per month | 2 | 15 | 8 | 1 | 2 |  | 13 | 12 | 5 | | 0 | 1 |  |
|  | Never | 3 | 22 | 6 | 4 | 0 |  | 13 | 17 | 2 | | 3 | 1 |  |

| Food group:  Fruits | | Control | | | | |  | Cases | | | | |  | |
| --- | --- | --- | --- | --- | --- | --- | --- | --- | --- | --- | --- | --- | --- | --- |
|  |  | Underweight | Normal Weight | Overweight | Obese I | Obese II | P-Value | Underweight | Normal Weight | Overweight | Obese I | Obese II | P-Value |  |
| Banana | Daily | 1 | 8 | 6 | 1 | 0 | 0.092 | 6 | 2 | 1 | 0 | 1 | 0.180 |  |
|  | Once/ week | 1 | 22 | 9 | 3 | 2 |  | 12 | 21 | 5 | 2 | 1 |  |  |
|  | 2-3/week | 10 | 28 | 17 | 7 | 3 |  | 19 | 20 | 2 | 2 | 0 |  |  |
|  | 2-3 per month | 5 | 23 | 10 | 4 | 0 |  | 18 | 26 | 11 | 4 | 2 |  |  |
|  | Never | 2 | 15 | 4 | 0 | 0 |  | 9 | 9 | 6 | 1 | 1 |  |  |
| Oranges | Daily | 1 | 15 | 9 | 3 | 0 | 0.469 | 10 | 10 | 2 | 2 | 1 | 0.451 |  |
|  | Once/ week | 3 | 25 | 8 | 3 | 1 |  | 14 | 22 | 5 | 0 | 1 |  |  |
|  | 2-3/week | 9 | 29 | 15 | 6 | 3 |  | 19 | 15 | 6 | 5 | 1 |  |  |
|  | 2-3 per month | 4 | 17 | 9 | 3 | 1 |  | 15 | 24 | 9 | 1 | 2 |  |  |
|  | Never | 2 | 10 | 5 | 0 | 0 |  | 6 | 7 | 3 | 1 | 0 |  |  |
| Mango | Daily | 1 | 7 | 4 | 1 | 0 | 0.475 | 5 | 4 | 1 | 1 | 1 | 0.776 |  |
|  | Once/ week | 2 | 19 | 6 | 3 | 2 |  | 10 | 18 | 3 | 1 | 0 |  |  |
|  | 2-3/week | 10 | 23 | 14 | 6 | 2 |  | 16 | 17 | 5 | 4 | 0 |  |  |
|  | 2-3 per month | 2 | 28 | 13 | 5 | 1 |  | 18 | 23 | 9 | 2 | 3 |  |  |
|  | Never | 4 | 19 | 9 | 0 | 0 |  | 15 | 16 | 7 | 1 | 1 |  |  |
| Grapefruit | Daily | 1 | 4 | 2 | 0 | 0 | 0.336 | 1 | 2 | 0 | 0 | 0 | 0.997 |  |
|  | Once/ week | 3 | 11 | 7 | 0 | 1 |  | 4 | 14 | 3 | 0 | 0 |  |  |
|  | 2-3/week | 4 | 11 | 11 | 1 | 2 |  | 15 | 11 | 2 | 2 | 0 |  |  |
|  | 2-3 per month | 3 | 21 | 13 | 8 | 0 |  | 8 | 18 | 8 | 3 | 0 |  |  |
|  | Never | 8 | 49 | 13 | 6 | 2 |  | 36 | 33 | 12 | 4 | 5 |  |  |
| Dates | Daily | 8 | 24 | 12 | 7 | 1 | 0.564 | 24 | 21 | 8 | 4 | 2 | 0.460 |  |
|  | Once/ week | 0 | 13 | 8 | 0 | 0 |  | 8 | 11 | 1 | 2 | 0 |  |  |
|  | 2-3/week | 5 | 34 | 14 | 4 | 4 |  | 20 | 24 | 11 | 1 | 1 |  |  |
|  | 2-3 per month | 3 | 18 | 11 | 3 | 0 |  | 7 | 15 | 3 | 1 | 2 |  |  |
|  | Never | 3 | 7 | 1 | 1 | 0 |  | 5 | 7 | 2 | 1 | 0 |  |  |
| Guuava | Daily | 1 | 7 | 3 | 1 | 0 | 0.915 | 3 | 6 | 1 | 0 | 0 | 0.469 |  |
|  | Once/ week | 3 | 13 | 7 | 0 | 2 |  | 7 | 7 | 3 | 0 | 0 |  |  |
|  | 2-3/week | 6 | 17 | 9 | 5 | 0 |  | 17 | 12 | 3 | 2 | 1 |  |  |
|  | 2-3 per month | 3 | 29 | 11 | 8 | 0 |  | 14 | 29 | 9 | 6 | 1 |  |  |
|  | Never | 6 | 30 | 16 | 1 | 3 |  | 23 | 24 | 9 | 1 | 3 |  |  |
| Lemon | Daily | 8 | 41 | 22 | 8 | 2 | 0.516 | 21 | 28 | 11 | 2 | 2 | 0.925 |  |
|  | Once/ week | 2 | 13 | 3 | 1 | 0 |  | 10 | 11 | 4 | 1 | 0 |  |  |
|  | 2-3/week | 4 | 24 | 14 | 4 | 2 |  | 17 | 17 | 5 | 4 | 1 |  |  |
|  | 2-3 per month | 4 | 3 | 4 | 1 | 0 |  | 7 | 7 | 1 | 0 | 0 |  |  |
|  | Never | 1 | 15 | 3 | 1 | 1 |  | 9 | 15 | 4 | 2 | 2 |  |  |
| Melon | Daily | 0 | 2 | 1 | 0 | 0 | 0.345 | 0 | 2 | 0 | 0 | 0 | 0.115 |  |
|  | Once/ week | 1 | 9 | 4 | 0 | 1 |  | 5 | 6 | 1 | 0 | 0 |  |  |
|  | 2-3/week | 3 | 11 | 5 | 3 | 1 |  | 9 | 4 | 4 | 0 | 0 |  |  |
|  | 2-3 per month | 8 | 44 | 24 | 9 | 2 |  | 28 | 37 | 11 | 5 | 0 |  |  |
|  | Never | 7 | 30 | 12 | 3 | 1 |  | 22 | 29 | 9 | 4 | 5 |  |  |

| Food group:  Drinks-Juices | | Control | | | | |  | Cases | | | | |  |
| --- | --- | --- | --- | --- | --- | --- | --- | --- | --- | --- | --- | --- | --- |
|  |  | Underweight | Normal Weight | Overweight | Obese I | Obese II | P-Value | Underweight | Normal Weight | Overweight | Obese I | Obese II | P-Value |
| Lemon drink | Daily | 7 | 29 | 16 | 7 | 2 | 0.248 | 20 | 18 | 8 | 2 | 1 | 0.558 |
|  | Once/ week | 3 | 12 | 8 | 4 | 0 |  | 7 | 12 | 5 | 1 | 1 |  |
|  | 2-3/week | 5 | 33 | 12 | 3 | 2 |  | 23 | 25 | 7 | 3 | 1 |  |
|  | 2-3 per month | 2 | 7 | 5 | 1 | 0 |  | 6 | 11 | 1 | 1 | 0 |  |
|  | Never | 2 | 15 | 5 | 0 | 1 |  | 8 | 12 | 4 | 2 | 2 |  |
| Hibiscus | Daily | 1 | 11 | 4 | 2 | 0 | 0.243 | 7 | 7 | 0 | 0 | 0 | 0.342 |
|  | Once/ week | 2 | 10 | 6 | 1 | 1 |  | 8 | 14 | 2 | 2 | 0 |  |
|  | 2-3/week | 7 | 12 | 9 | 5 | 2 |  | 14 | 14 | 6 | 1 | 1 |  |
|  | 2-3 per month | 1 | 17 | 10 | 3 | 1 |  | 10 | 17 | 6 | 3 | 2 |  |
|  | Never | 8 | 46 | 17 | 4 | 1 |  | 25 | 26 | 11 | 3 | 2 |  |
| Boabab | Daily | 0 | 2 | 1 | 0 | 0 | 0.770 | 1 | 4 | 0 | 0 | 0 | 0.221 |
|  | Once/ week | 2 | 2 | 2 | 0 | 0 |  | 0 | 3 | 0 | 0 | 0 |  |
|  | 2-3/week | 2 | 3 | 3 | 0 | 0 |  | 1 | 5 | 3 | 0 | 0 |  |
|  | 2-3 per month | 3 | 6 | 8 | 1 | 1 |  | 6 | 7 | 3 | 1 | 1 |  |
|  | Never | 12 | 83 | 32 | 14 | 4 |  | 56 | 59 | 19 | 8 | 4 |  |
| Bottled drinks | Daily | 3 | 13 | 5 | 0 | 0 | 0.115 | 7 | 5 | 0 | 0 | 0 | 0.015 |
|  | Once/ week | 3 | 16 | 9 | 2 | 0 |  | 11 | 11 | 2 | 2 | 0 |  |
|  | 2-3/week | 4 | 20 | 5 | 2 | 2 |  | 11 | 10 | 5 | 0 | 1 |  |
|  | 2-3 per month | 3 | 15 | 6 | 5 | 2 |  | 12 | 19 | 3 | 4 | 0 |  |
|  | Never | 6 | 32 | 21 | 6 | 1 |  | 23 | 33 | 15 | 3 | 4 |  |

| Food group:  Vegetables | | Control | | | | |  | Cases | | | | |  |
| --- | --- | --- | --- | --- | --- | --- | --- | --- | --- | --- | --- | --- | --- |
|  |  | Underweight | Normal Weight | Overweight | Obese I | Obese II | P-Value | Under  weight | Normal Weight | Overweight | Obese I | Obese II | P-Value |
| Fresh-Mixed-Salad | Daily | 9 | 49 | 27 | 11 | 4 | 0.262 | 29 | 34 | 15 | 5 | 2 | 0.736 |
|  | Once/ week | 4 | 14 | 2 | 0 | 0 |  | 12 | 14 | 0 | 1 | 2 |  |
|  | 2-3/week | 6 | 29 | 12 | 4 | 1 |  | 19 | 22 | 7 | 2 | 1 |  |
|  | 2-3 per month | 0 | 2 | 4 | 0 | 0 |  | 2 | 4 | 2 | 1 | 0 |  |
|  | Never | 0 | 2 | 1 | 0 | 0 |  | 2 | 4 | 1 | 0 | 0 |  |
| Cooked-vegetables | Daily | 6 | 22 | 20 | 10 | 1 | 0.005 | 12 | 28 | 11 | 4 | 1 | 0.018 |
|  | Once/ week | 2 | 14 | 6 | 1 | 1 |  | 13 | 11 | 2 | 1 | 1 |  |
|  | 2-3/week | 6 | 32 | 11 | 3 | 3 |  | 17 | 17 | 7 | 3 | 3 |  |
|  | 2-3 per month | 2 | 7 | 3 | 0 | 0 |  | 6 | 7 | 2 | 0 | 0 |  |
|  | Never | 3 | 21 | 6 | 1 | 0 |  | 16 | 15 | 3 | 1 | 0 |  |
| Onions | Daily | 13 | 65 | 36 | 11 | 3 | 0.467 | 39 | 49 | 18 | 8 | 3 | 0.287 |
|  | Once/ week | 3 | 6 | 1 | 0 | 0 |  | 9 | 9 | 2 | 0 | 0 |  |
|  | 2-3/week | 2 | 14 | 7 | 3 | 2 |  | 9 | 8 | 2 | 1 | 2 |  |
|  | 2-3 per month | 0 | 2 | 1 | 0 | 0 |  | 2 | 6 | 1 | 0 | 0 |  |
|  | Never | 1 | 9 | 1 | 1 | 0 |  | 5 | 6 | 2 | 0 | 0 |  |
| Tomatoes | Daily | 11 | 47 | 26 | 10 | 4 | 0.625 | 31 | 32 | 17 | 5 | 0 | 0.845 |
|  | Once/ week | 5 | 12 | 4 | 0 | 0 |  | 10 | 10 | 1 | 1 | 0 |  |
|  | 2-3/week | 2 | 29 | 10 | 4 | 1 |  | 16 | 25 | 6 | 2 | 2 |  |
|  | 2-3 per month | 0 | 3 | 3 | 1 | 0 |  | 1 | 5 | 0 | 1 | 3 |  |
|  | Never | 1 | 5 | 3 | 0 | 0 |  | 6 | 6 | 1 | 0 | 0 |  |
| Cucumber | Daily | 8 | 36 | 28 | 10 | 4 | 0.003 | 25 | 26 | 14 | 5 | 2 | 0.268 |
|  | Once/ week | 4 | 11 | 2 | 0 | 0 |  | 10 | 14 | 1 | 2 | 1 |  |
|  | 2-3/week | 2 | 25 | 10 | 5 | 1 |  | 15 | 22 | 5 | 1 | 1 |  |
|  | 2-3 per month | 1 | 7 | 4 | 0 | 0 |  | 0 | 6 | 3 | 0 | 0 |  |
|  | Never | 4 | 17 | 2 | 0 | 0 |  | 14 | 10 | 2 | 1 | 1 |  |
| Carrot | Daily | 6 | 32 | 23 | 7 | 4 | 0.053 | 12 | 15 | 10 | 4 | 1 | 0.143 |
|  | Once/ week | 5 | 12 | 5 | 1 | 0 |  | 7 | 9 | 3 | 1 | 1 |  |
|  | 2-3/week | 4 | 27 | 7 | 6 | 1 |  | 19 | 18 | 7 | 2 | 0 |  |
|  | 2-3 per month | 3 | 7 | 7 | 0 | 0 |  | 9 | 12 | 0 | 0 | 1 |  |
|  | Never | 1 | 18 | 4 | 1 | 0 |  | 17 | 24 | 5 | 2 | 2 |  |
| Rocket-plant | Daily | 4 | 19 | 10 | 4 | 1 | 0.851 | 10 | 7 | 5 | 2 | 1 | 0.776 |
|  | Once/ week | 2 | 11 | 2 | 2 | 1 |  | 8 | 9 | 1 | 0 | 1 |  |
|  | 2-3/week | 3 | 16 | 5 | 2 | 0 |  | 9 | 13 | 5 | 1 | 1 |  |
|  | 2-3 per month | 1 | 4 | 4 | 1 | 0 |  | 7 | 6 | 2 | 1 | 0 |  |
|  | Never | 9 | 46 | 25 | 6 | 3 |  | 30 | 43 | 12 | 5 | 2 |  |
| Okra | Daily | 3 | 7 | 2 | 2 | 1 | 0.891 | 7 | 3 | 2 | 1 | 0 | 0.667 |
|  | Once/ week | 3 | 27 | 8 | 4 | 2 |  | 14 | 24 | 3 | 4 | 2 |  |
|  | 2-3/week | 5 | 35 | 21 | 5 | 1 |  | 21 | 26 | 9 | 3 | 3 |  |
|  | 2-3 per month | 3 | 19 | 8 | 3 | 0 |  | 9 | 16 | 6 | 1 | 0 |  |
|  | Never | 5 | 8 | 7 | 1 | 1 |  | 13 | 9 | 5 | 0 | 0 |  |
| Jew-mallow | Daily | 0 | 1 | 0 | 0 | 0 | 0.322 | 1 | 0 | 0 | 0 | 0 | 0.989 |
|  | Once/ week | 4 | 17 | 7 | 2 | 1 |  | 11 | 18 | 5 | 1 | 1 |  |
|  | 2-3/week | 5 | 20 | 13 | 6 | 2 |  | 15 | 18 | 6 | 5 | 0 |  |
|  | 2-3 per month | 2 | 21 | 11 | 5 | 1 |  | 14 | 17 | 3 | 1 | 1 |  |
|  | Never | 8 | 37 | 15 | 2 | 1 |  | 23 | 25 | 11 | 2 | 3 |  |
| Egg-plant | Daily | 0 | 0 | 1 | 1 | 0 | 0.499 | 1 | 0 | 0 | 0 | 0 | 0.260 |
|  | Once/ week | 4 | 21 | 7 | 2 | 2 |  | 11 | 22 | 3 | 0 | 1 |  |
|  | 2-3/week | 5 | 16 | 7 | 6 | 1 |  | 14 | 9 | 6 | 2 | 1 |  |
|  | 2-3 per month | 2 | 21 | 12 | 4 | 0 |  | 17 | 21 | 6 | 2 | 0 |  |
|  | Never | 8 | 38 | 19 | 2 | 2 |  | 21 | 26 | 10 | 5 | 3 |  |
| Potatoes | Daily | 7 | 7 | 3 | 3 | 1 | 0.236 | 7 | 5 | 3 | 1 | 1 | 0.821 |
|  | Once/ week | 5 | 21 | 9 | 3 | 1 |  | 14 | 23 | 7 | 1 | 2 |  |
|  | 2-3/week | 5 | 51 | 23 | 8 | 3 |  | 26 | 30 | 9 | 3 | 1 |  |
|  | 2-3 per month | 1 | 10 | 6 | 1 | 0 |  | 12 | 15 | 4 | 2 | 1 |  |
|  | Never | 1 | 7 | 5 | 0 | 0 |  | 5 | 5 | 2 | 2 | 0 |  |
