# Supplementary material for: The nutritional status of mycetoma affected patients seen at the Mycetoma Research Center, Sudan
Source: PLoS Negl Trop Dis. 2024 Jan 2;18(1):e0011726. doi: 10.1371/journal.pntd.0011726 (PMC10786388; doi:10.1371/journal.pntd.0011726)
Supplement: Attachment — Submitted filename: response to Reviewer.docx [file pntd.0011726.s003.docx]

**Reviewer's Responses to Questions**

Reviewer #1: The objectives are clear. But I have some queries.

How was the family member from the control group chosen?

- A convenience sampling technique was used in this study. Line 139

Were the controls paired by age?

- Indeed, controls were paired by age. Line 28,

In case no family member was present, how was that control chosen?

- In general, due to the extended family culture in Sudan, all patients come to the clinic with a family member living in the same house, and in general, all of them share the same type of food.

Was that patient excluded from the analysis if no family member was available?

- That was not done.

How was the nutritional interview performed? Was a specific format or validated questionnaire employed? Please include this in the methods section.

Stated in lines 144-148

Please include in the methods section how the “appetite” was determined.

- Patients and control participants assessed their appetite, categorising it as low, moderate, or high based on their personal opinions. While this evaluation is subjective, it reflects their individual beliefs and perceptions.

Reviewer #1: In the results section,

please include kg and cm where appropriate.

- Done

I believe the last paragraph of the result section could be improved.

- Done

I don’t think it is necessary to present some foods such as cucumber, carrot, and sunflower oil consumption as independent variables. I believe it would be better to group certain food groups and analyse them together (example: vegetables, fruits, etc).

- There is a lot of data obtained, and we found it difficult not to show that.

Reviewer #1: The discussion is well-presented and written. The limitations are clearly stated.

I would add that body composition by bioelectrical impedance analysis is needed too

- Done

the conclusion is valid. I would modify this sentence:

“The successful intake of probiotics as ingestible adjuvants and immune modulators for antiviral immunity and management of SARS-CoV-2 infection and COVID-19 is worth studying in mycetoma.” as the evidence of their usefulness in COVID-19 is poor.

- Done

Reviewer #1: The authors present an interesting study of nutrition in patients with mycetoma from Sudan. The methods section could be improved by thoroughly explaining the control group and the nutritional questionnaire employed. The article is generally well-written and information about nutritional status in patients with mycetoma is very scarce.
