# Supplementary material for: The nutritional status of mycetoma affected patients seen at the Mycetoma Research Center, Sudan
Source: PLoS Negl Trop Dis. 2024 Jan 2;18(1):e0011726. doi: 10.1371/journal.pntd.0011726 (PMC10786388; doi:10.1371/journal.pntd.0011726)
Supplement: Attachment — Submitted filename: Response to Reviewer -2.docx [file pntd.0011726.s004.docx]

Reviewer #1: The authors have improved the manuscript. Some corrections are still needed.

The abstract needs extensive modification. The authors should include, not only results, but some of the conclusions that are present in the main manuscript. Please improve the abstract by including the most relevant results of your analysis.

The abstract was revised, and only important results were included.

The sentence “The Body Mass Index (BMI) for the study population was calculated, revealing that 43.3% of the patients and 50.3% of the control had BMI.” is incorrect. Please correct it.

It is correct

The previous query “I don’t think it is necessary to present some foods such as cucumber, carrot, and sunflower oil consumption as independent variables. I believe it would be better to group certain food groups and analyze them together (example: vegetables, fruits, etc)” was responded by the authors as:

“There is a lot of data obtained, and we found it difficult not to show that. “

Please justify including variables like “cucumber and carrot consumption” as independent variables and include a justification for this and its relevance in the discussion.

That was deleted.

Only important results were included in the results section.

--------------------


 
