# Supplementary material for: The nutritional status of mycetoma affected patients seen at the Mycetoma Research Center, Sudan
Source: PLoS Negl Trop Dis. 2024 Jan 2;18(1):e0011726. doi: 10.1371/journal.pntd.0011726 (PMC10786388; doi:10.1371/journal.pntd.0011726)
Supplement: Attachment — Submitted filename: Letter to Editor 3.docx [file pntd.0011726.s005.docx]

Dear Editor

Thank you very much indeed for your kind note, and apologies for this typo mistake.

All the required corrections were done and highlighted in yellow.

Best regards

Prof Fahal on behalf of the authors
