# Supplementary material for: The nutritional status of mycetoma affected patients seen at the Mycetoma Research Center, Sudan
Source: PLoS Negl Trop Dis. 2024 Jan 2;18(1):e0011726. doi: 10.1371/journal.pntd.0011726 (PMC10786388; doi:10.1371/journal.pntd.0011726)
Supplement: Attachment — Submitted filename: letter to the editor 4.docx [file pntd.0011726.s006.docx]

Dear Editor

The authors corrected the figurers in lines 35 and 175, and in table no. 3, line 184 to 11%.

Best regards

Prof Fahal

On behalf of the authors
